# Synergistic anti-SARS-CoV-2 activity of repurposed anti-parasitic drug combinations

**DOI:** 10.1186/s40360-022-00580-8

**Published:** 2022-06-18

**Authors:** Kunlakanya Jitobaom, Chompunuch Boonarkart, Suwimon Manopwisedjaroen, Nuntaya Punyadee, Suparerk Borwornpinyo, Arunee Thitithanyanont, Panisadee Avirutnan, Prasert Auewarakul

**Affiliations:** 1 Department of Microbiology, Faculty of Medicine Siriraj Hospital, Mahidol University, Bangkok, 10700 Thailand; 2 Department of Microbiology, Faculty of Science, Mahidol University, Bangkok, 10400 Thailand; 3 Division of Dengue Hemorrhagic Fever Research, Department of Research and Development, Faculty of Medicine Siriraj Hospital, Mahidol University, Bangkok, 10700 Thailand; 4 Siriraj Center of Research Excellence in Dengue and Emerging Pathogens, Faculty of Medicine Siriraj Hospital, Mahidol University, Bangkok, 10700 Thailand; 5 Department of Biotechnology, Faculty of Science, Mahidol University, Bangkok, 10400 Thailand

**Keywords:** SARS-CoV-2, Repurposed drug, Anti-parasitic drugs, Niclosamide, Ivermectin, Chloroquine

## Abstract

**Background:**

COVID-19 pandemic has claimed millions of lives and devastated the health service system, livelihood, and economy in many countries worldwide. Despite the vaccination programs in many countries, the spread of the pandemic continues, and effective treatment is still urgently needed. Although some antiviral drugs have been shown to be effective, they are not widely available. Repurposing of anti-parasitic drugs with in vitro anti-SARS-CoV-2 activity is a promising approach being tested in many clinical trials. Combination of these drugs is a plausible way to enhance their effectiveness.

**Methods:**

The in vitro anti-SARS-CoV-2 activity of combinations of niclosamide, ivermectin and chloroquine were evaluated in Vero E6 and lung epithelial cells, Calu-3.

**Results:**

All the two-drug combinations showed higher potency resulting in up to 4-fold reduction in the half maximal inhibitory concentration (IC_50_) values compared to individual drugs. Among these combinations, niclosamide-ivermectin achieved the highest inhibitory level of over 99%. Combination synergy analysis showed niclosamide-ivermectin combination to have the best synergy score with a mean Loewe synergy score of 4.28 and a peak synergy score of 24.6 in Vero E6 cells and a mean Loewe synergy score of 3.82 and a peak synergy score of 10.86 in Calu-3 cells.

**Conclusions:**

The present study demonstrated the benefit of drug combinations on anti-SARS-CoV-2 activity. Niclosamide and ivermectin showed the best synergistic profile and should be further tested in clinical trials.

**Supplementary Information:**

The online version contains supplementary material available at 10.1186/s40360-022-00580-8.

## Background

The spread of SARS-CoV-2 and the COVID-19 pandemic has swept through countries and continents causing catastrophic loss of lives, public health, livelihood, and economy. Up to March 2021, more than hundred million cases have been reported with over two million deaths [1]. The hope to get through the pandemic and resume normal life relies heavily on vaccine deployment, which will still take months or years in most less-developed countries [2]. One of the reasons for the heavy loss of lives, hospital overload, and public panic is the lack of effective treatment. Remdesivir is now the only antiviral drug with emergency use authorization by US FDA [3]. The drug is, however, not yet widely available. Other FDA-approved drugs are anti-inflammatory targeting host inflammatory responses [4]. More drugs capable of inhibiting SARS-CoV-2 replication are urgently needed not only for treatment but also for reducing viral load and transmission [5]. Many repurposed anti-parasitic drugs have been shown to possess in vitro activity against SARS-CoV-2.

In vitro screenings of FDA-approved drugs have identified a number of anti-parasitic drugs with anti-SARS-CoV-2 activity and potential for drug repurposing for treatment of COVID-19 patients [6, 7]. The early hope to get an effective treatment using these drugs was let down by the failure to show clinical benefit of chloroquine in clinical trials [8]. On the other hand, ivermectin has shown promising results in some clinical trials [9–13]. Ivermectin has been shown to cause up to 5000-fold reduction in SARS-CoV-2 replication in vitro [14–16]. The drug has been widely used to treat various parasitic diseases in humans and animals for four decades with little safety concern. It was also used in the mass treatment campaign against river blindness (Onchocerciasis) with good safety record [17]. It is therefore, an attractive option for drug repurposing for COVID-19 treatment. Another anti-parasitic drug, niclosamide, showed a good anti-SARS-CoV-2 activity with a high selective index [7, 18]. The drug has been shown to exhibit broad antiviral activity against a wide range of viruses [19]. These anti-parasitic drugs with potent in vitro anti-SARS-CoV-2 activity are widely available, inexpensive, and considered relatively safe for short-term usage.

The world urgently needs repurposed drug regimens with higher antiviral activity against SARS-CoV-2 to cope with the pandemic. One of the approaches to enhance drug potency is through drug combination. To find a drug combination with good therapeutic potential, we tested combinations of these common drugs for in vitro synergistic activity against SARS-CoV-2.

## Methods

### Chemicals

All drugs were prepared to 10 mM stock solutions in 100% DMSO (Sigma) for niclosamide (N3510, Sigma) and ivermectin (I8898, Sigma), or water for chloroquine (HY-17589, MCE) and stored at − 80 °C. The drugs were diluted to the working concentrations in 2%FBS-MEM or 2%FBS-DMEM/F12 for the experiments in Vero E6 or Calu-3 cells, respectively. The final concentration of DMSO was 0.5% in all experiments.

### Cells and viruses

Calu-3 cells were obtained from ATCC, USA (Cat. No. HTB-55). The cells were cultivated in Dulbecco’s Modified Eagle Medium/Nutrient Mixture F-12 (DMEM/F-12; 11320033, Gibco) supplemented with 10% heat inactivated FBS at 37 °C with 5% CO_2_. Vero E6 cells (Vero C1008) were obtained from ATCC, USA (Cat. No. CRL-1586). The cells were cultivated in the minimum essential medium (MEM; 10–009-CV, Corning) supplemented with 10% heat inactivated FBS at 37 °C with 5% CO_2_.

SARS-CoV-2 (SARS-CoV-2/01/human/Jan2020/Thailand) representing the original Wuhan strain was isolated from nasopharyngeal swabs of a COVID-19 patient in Thailand in the previous study [20]. The protein sequence of surface glycoprotein of SARS-CoV-2/01/human/Jan2020/Thailand is available at GenBank: QYZ85362.1. Vero E6 cells was used for viral propagation. The supernatants containing virus were harvested when 50% of the infected cells display cytopathic effect (CPE). Centrifugation was performed to remove cell debris. The virus supernatants were aliquoted and stored at -80 °C as a virus stock.

### Viral quantifications

#### Plaque assay for SARS-CoV-2

Vero E6 cells were plated in 24-well plates at a density of 1.3 × 10^5^ cells per well, which allowed 100% confluence to be reached within 24 hours. The culture medium was removed and 300 μl of serum free-MEM was added. After that, the cells were incubated with 10-fold serial dilution of virus supernatants for 1 hour at 37 °C with 5%CO_2_. Subsequently, the virus inoculums were removed, and the cells were immediately overlaid with 1 ml of 1.56% microcrystalline cellulose (Avicel, RC-591) in 2%FBS-MEM. The infected cells were further incubated in the standard condition for 3 days. At 3 days after infection, the overlaid media were removed. The infected cells were fixed with 10% (v/v) formalin in phosphate-buffered saline for 2 hours. After that, fixed cells were washed in tap water and stained with 1% (w/v) crystal violet in 20% (v/v) ethanol for 5 min. The excess dyes were removed by washing in tap water. The titers of virus were calculated in plaque forming units per ml (pfu/ml).

#### 50% tissue culture infectious dose (TCID_50_) endpoint dilution assay coupled with ELISA

Before the day of infection, Calu-3 cells were seeded at a density of 2.0 × 10^4^ cells/well in 96-well plates. Next day, the culture medium was removed, and the cells were inoculated with 100 μl of half-log10 serial dilution of the virus supernatants for 2 days in the standard condition. Subsequently, the supernatants were discarded, and the infected cells were fixed with the mixture of absolute methanol and acetone in 1:1 ratio for 30 min at 4 °C. The infected cells were detected based on ELISA assay using the antibody against the SARS-CoV-2 nucleocapsid protein (NP) (40143-R001, Sino Biological) and anti-rabbit IgG-conjugated HRP. The TCID_50_ titers were calculated following the Reed and Muench method [21].

#### One-step quantitative reverse-transcription PCR (qRT-PCR)

In this study, one-step qRT-PCR was used as a screening assay to detect the RNA of SARS-CoV-2 directly from the virus supernatants. The procedure was previously described elsewhere [22]. For the sample preparation, the virus supernatants were subjected to disinfection by heat inactivation at 70 °C for 20 min. Then the heat inactivated virus supernatants were diluted for a 1:10 ratio in DNase/RNase free distilled water. Subsequently, one-step qRT-PCR was performed using the Power SYBR one-step kit (Applied Biosystems) in the LightCycler 480 (Roche, LC480) following the kit’s instructions for a 10 μl reaction volume. The forward and reverse primers used in this study were N-Fw: 5′-GGGGAACTTCTCCTGCTAGAAT-3’and N-Rv: 5′-CAGACATTTTGCTCTCAAGCTG-3′, respectively. TRIzol-LS (Invitrogen) purified RNA of SAR-CoV-2 stock virus was used as a positive control. The nuclease-free water and mock-infected supernatants were used as no-template control.

The thermocycler was run following the instructions of Power SYBR one-step kit. The reverse transcription and the activation of polymerase were performed at 48 °C for 30 min and 95 °C for 10 min, respectively. The amplification step was performed for 45 cycles at 95 °C for 15 s, 60 °C for 1 min and the melting curve step at 95 °C for 30s, 60 °C for 30s. The Abs Quant/2nd derivative method was used to calculate threshold cycle (Ct) values. The melting temperature (T_m_) of the PCR products were analyzed and compared with the product amplified from positive control to exclude the reactions with non-specific amplification. The percent inhibition was calculated relative to the cells treated with 0.5% DMSO.

#### Cell viability assay

Vero E6 or Calu-3 cells were seeded in 96 well-plates at a density of 2.5 × 10^4^ or 2.0 × 10^4^ cells per well, respectively. Then niclosamide, ivermectin, and chloroquine at various concentrations in 2%FBS-MEM or 2%FBS-DMEM/F12 were added to Vero E6 or Calu-3 cells, respectively, for 48 hours. Subsequently, the cell viability was assessed using MTT dyes (Invitrogen) in duplicate (Supplementary file 1). The procedure was described elsewhere [23]. The viability of drug-treated cells was expressed as percent cell viability relative to 0.5% DMSO-treated cells.

### Antiviral activity against SARS-CoV-2 in vitro

#### Single drug treatments

Vero E6 or Calu-3 cells were seeded in 96 well-plates at a density of 2.5 × 10^4^ or 2.0 × 10^4^ cells per well, respectively. The cultured medium was removed, and the cells were incubated with twofold serially diluted drugs in 2%FBS-media for 1 hour at 37 °C with 5% CO_2_. The medium containing 0.5% DMSO was used as no drug control. After that, the Vero E6 or Calu-3 cells were inoculated with SARS-CoV-2 at multiplicity of infection (MOI) 0.01 or 500 TCID_50_, respectively, for 1 hour. Then the inoculum was discarded, and the cells were further maintained in the media containing drugs at various indicated concentrations or 0.5%DMSO. The virus supernatants were collected at 48 hours after infection. The virus titers were determined using both plaque assay and one-step qRT-PCR. The experiments were repeated at least three times (Supplementary file 1).

#### Two-drug combinations treatments

The experiments were performed according to the single drug treatment protocol. Except the cells were treated for 1 hour with 16 different pairwise combinations of two drugs. Four concentrations of each single drug were used which are at 2×, 1×, 0.5×, and 0.25× of IC_50_ values that were evaluated from the single drug treatments. The experiments were repeated at least three times (Supplementary file 1). The cell viability was also assessed using MTT dyes as mentioned earlier.

#### The combination synergy analysis

The combination synergy of two-drug combinations was analyzed using SynergyFinderPlus (www.synergyfinderplus.org) [24]. Four reference models were used in this study, including the Loewe additivity (Loewe) [25], Zero Independence Potency (ZIP), Highest Single Agent (HSA), and Bliss independence models.

### Statistical analysis

The independent experiments were performed in triplicate, and data are shown as mean ± SD. The 50% cytotoxic concentration (CC_50_) and the half-maximal inhibitory concentration (IC_50_) were calculated from the dose-response curves of drug treatment by non-linear regression analysis using GraphPad Prism 8 (GraphPad Software, Inc., CA).

## Results

### Evaluation of single drug treatment against SARS-CoV-2 in Vero E6 cells

**Fig. 1:**
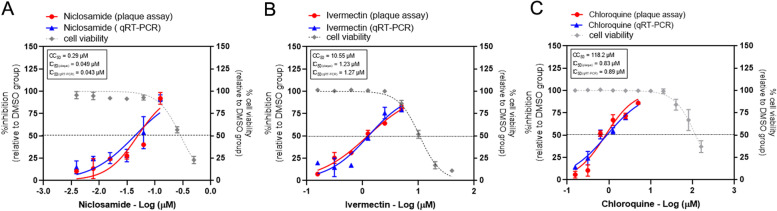
Evaluation of antiviral activity of drug candidates against SARS-CoV-2 in vitro*.* The dose-response curves of single drug treatments against SARS-CoV-2 are shown; (**A**) niclosamide, (**B**) ivermectin, and (**C**) chloroquine. Vero E6 cells were treated with various concentrations of drug for 1 hour and followed by SARS-CoV-2 infection at MOI of 0.01. After removing of virus, the cells were maintained in the medium containing drugs or 0.5%DMSO for 2 days. The virus supernatants were collected for titration using the plaque assay and one step-qRT-PCR. The dose-response curves were expressed as the percent inhibition in relative to DMSO-treated cell control. The effect of drug treatment on the cell viability was determined using MTT assay and is expressed in relative to the DMSO-treated cell control. The experiments were repeated at least three times, and data are shown as mean ± SD

Fig. 1 and Table 1 show the anti-SARS-CoV-2 activities and cytotoxicity of the repurposed drugs in Vero E6 cells. The plaque assay was used to determine the viral production and is expressed as the percent inhibition relative to the viral titer of DMSO-treated cells. The one-step qRT-PCR was used to quantitate the viral RNA in virus supernatants and is also expressed as the percent inhibition relative to the DMSO-treated cells. The IC_50_ values calculated from the dose-response determined by plaque assay for niclosamide, ivermectin, and chloroquine were 0.049, 1.23, 0.046 and 0.83 μM, respectively. The IC_50_ values calculated from the dose-response determined by one-step qRT-PCR for niclosamide, ivermectin, and chloroquine were 0.043, 1.27, and 0.89 μM, respectively. Both methods used for viral quantification resulted in similar IC_50_ values. Thus, the viral RNA quantification by the one-step qRT-PCR accurately determined the infectious virus output in these experiments and could be used for the further two-drug combination experiments for the high throughput screening.

**Table 1:** Single drug treatment against SARS-CoV-2 in vitro

Drug candidates	Drug class	Drug indication	CC_**50**_ (μM)	IC_**50**_ (μM) Plaque assay	IC_**50**_ (μM) qRT-PCR
Niclosamide	Anthelminthic agents	Treatment of tapeworm and intestinal fluke infections	0.29	0.049	0.043
Ivermectin	Anti-parasitic agents	Treatment of onchocerciasis, and other worm infestations	10.55	1.23	1.27
Chloroquine	Anti-malarial agents	Treatment of malaria, rheumatic diseases and Zika virus infection	118.20	0.83	0.89

### Evaluation of two-drug combination treatments against SARS-CoV-2 in Vero E6 cells

Firstly, the antiviral activities of two-drug combinations were assessed in vitro in Vero E6 cells. The cells were treated with 16 different pairwise combinations of two drugs, including, niclosamide-ivermectin, niclosamide-chloroquine and ivermectin-chloroquine.

### Niclosamide-ivermectin combination

**Fig. 2:**
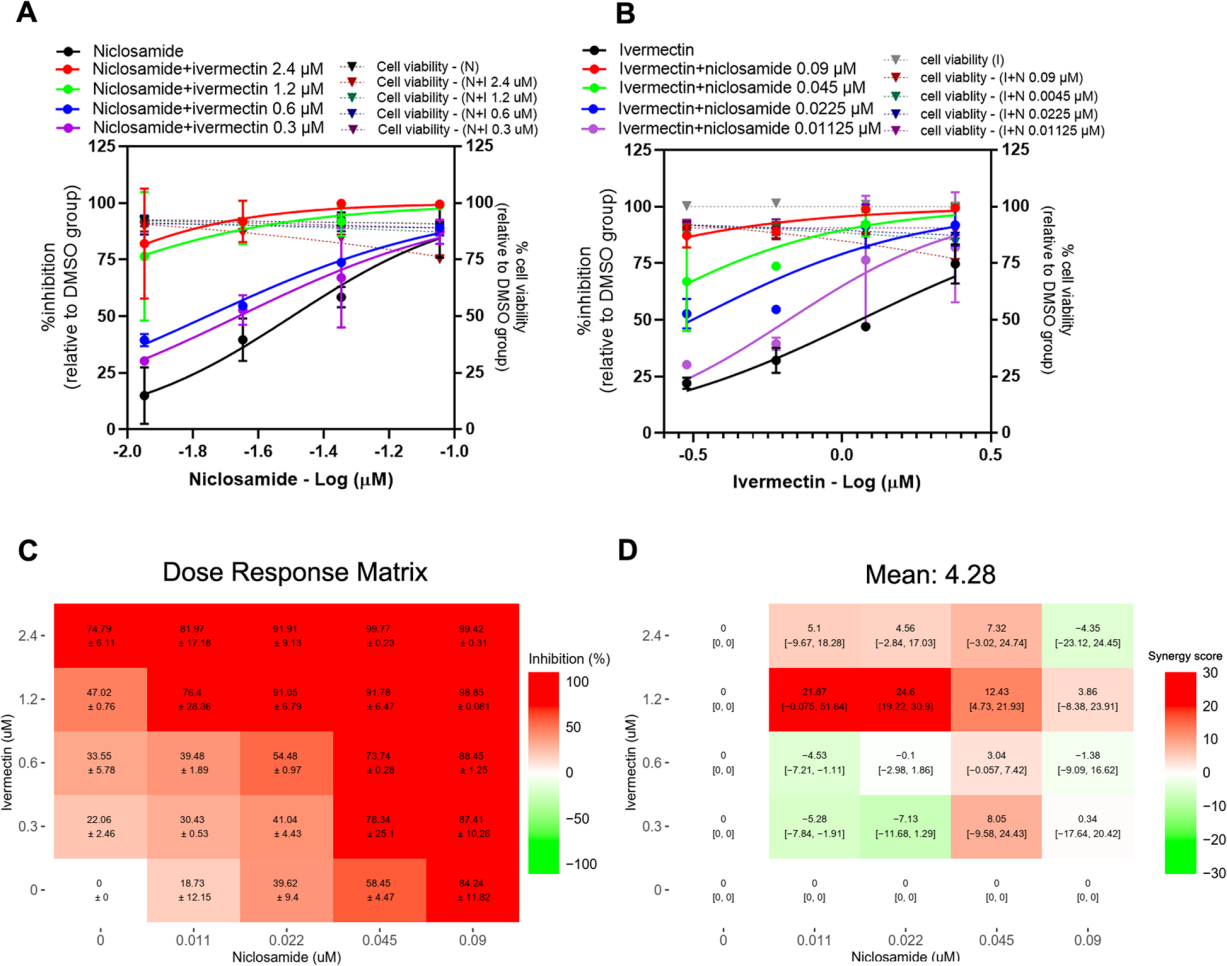
Niclosamide-ivermectin combination treatments against SARS-CoV-2 in Vero E6 cells. Vero E6 cells were treated for 1 hour with 16 different pairwise combinations of niclosamide and ivermectin. After that, the cells were infected with SARS-CoV-2 at MOI 0.01 for 1 hour. The virus inoculum was discarded, and the cells were further maintained in the medium containing drugs for 2 days. The viral RNA was quantitated using one-step qRT-PCR. The dose-response curves of two-drug combination treatments against SARS-CoV-2 are shown; (**A**) serial dilutions of niclosamide in the presence of different fixed concentrations of ivermectin, (**B**) serial dilutions of ivermectin in the presence of different fixed concentrations of niclosamide. The synergy scores of two-drug combinations were calculated using SynergyFinderPlus. The dose-response matrix (**C**) and the Loewe synergy score map of two-drug combination treatment (**D**) are shown. The synergy scores less than − 10 accounted for the antagonistic effect; from − 10 to 10 accounted for the additive effect; and larger than 10 accounted for the synergistic effect between two drugs. The experiments were repeated at least three times, and data are shown as mean ± SD in **A**, **B** and **C** or mean [95% confidence intervals (CI)] in **D**

The presence of ivermectin induced a shift in the dose-response curve of niclosamide, with approximately 2-fold reduction of niclosamide IC_50_ value in the presence of 0.6 and 0.3 μM ivermectin (Fig. 2A, Table 2). In a similar way, the presence of 0.0225 μM and 0.01125 μM niclosamide resulted in 4.06 and 1.92-fold reduction of ivermectin IC_50_ value, respectively (Fig. 2B, Table 2). The dose-response matrix of niclosamide and ivermectin combination showed the obvious increasing inhibitory effects with the maximal inhibitory activity of over 99% at the concentrations of 2-fold of the individual drug IC_50_ (Fig. 2C). From Fig. 2D, a synergy score plot shows positive Loewe synergy scores in the combinations with 1.2 μM and 2.4 μM ivermectin. Moreover, the combination of 0.0225 μM niclosamide and 1.2 μM ivermectin shows a peak Loewe synergy of 24.6, 95% confidence intervals (CI) [19.22, 30.9], which indicated a strong synergistic effect. The scores were slightly negative in the other part of the plot with lower ivermectin concentration indicating only additive effect at these lower concentrations. The mean Loewe synergy score is 4.28, which accounted for the additive effects between niclosamide and ivermectin in Vero E6 cells. Similar synergy scores of 3.97 and 4.26 were obtained using ZIP and Bliss independence reference models, respectively. The synergy scores calculated using HSA model was 16.02, which indicated a synergistic effect between niclosamide and ivermectin. No significant cytotoxicity in all 16 pairwise combinations (Fig. 2A, B).

**Table 2:** Antiviral activity of two-drug combinations treatment against SARS-CoV-2 in Vero E6 cells

Drug treatment	IC_**50**_ (μM) qRT-PCR	Fold reduction of IC_**50**_ (single/combined)
**Niclosamide-ivermectin**		
**Niclosamide**	**0.043**	
Niclosamide + ivermectin 2.4 μM	ND	ND
Niclosamide + ivermectin 1.2 μM	ND	ND
Niclosamide + ivermectin 0.6 μM	0.018	2.399
Niclosamide + ivermectin 0.3 μM	0.022	1.955
**Ivermectin**	**1.27**	
Ivermectin + niclosamide 0.09 μM	ND	ND
Ivermectin + niclosamide 0.0045 μM	ND	ND
Ivermectin + niclosamide 0.0225 μM	0.313	4.06
Ivermectin + niclosamide 0.01125 μM	0.660	1.92
**Niclosamide-chloroquine**		
**Niclosamide**	**0.043**	
Niclosamide + chloroquine 1.7 μM	ND	ND
Niclosamide + chloroquine 0.85 μM	ND	ND
Niclosamide + chloroquine 0.425 μM	0.013	3.308
Niclosamide + chloroquine 0.2125 μM	0.029	1.483
**Chloroquine**	**0.89**	
Chloroquine + niclosamide 0.09 μM	ND	ND
Chloroquine + niclosamide 0.0045 μM	ND	ND
Chloroquine + niclosamide 0.0225 μM	0.249	3.57
Chloroquine + niclosamide 0.01125 μM	0.531	1.68
**Ivermectin-chloroquine**		
**Ivermectin**	**1.27**	
Ivermectin + chloroquine 1.7 μM	ND	ND
Ivermectin + chloroquine 0.85 μM	ND	ND
Ivermectin + chloroquine 0.425 μM	0.515	2.47
Ivermectin + chloroquine 0.2125 μM	0.821	1.55
**Chloroquine**	**0.89**	
Chloroquine + ivermectin 2.4 μM	ND	ND
Chloroquine + ivermectin 1.2 μM	ND	ND
Chloroquine + ivermectin 0.6 μM	0.315	2.83
Chloroquine + ivermectin 0.3 μM	0.514	1.73

*ND* not determined, cannot calculate IC_50_ with the least curve fit of the data sets

### Niclosamide-chloroquine combination

**Fig. 3:**
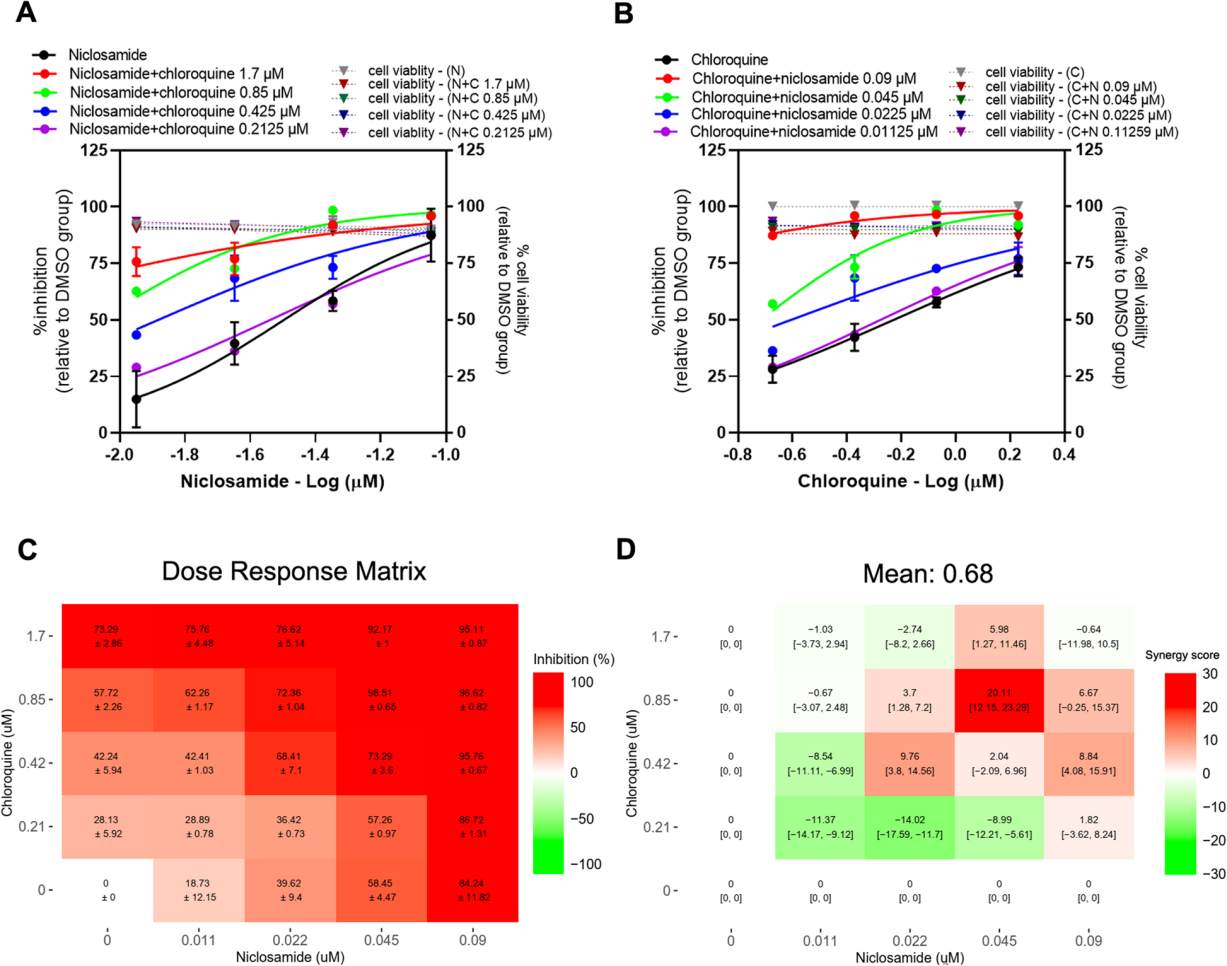
Niclosamide-chloroquine combination treatments against SARS-CoV-2 in Vero E6 cells. Vero E6 cells were treated for 1 hour with 16 different pairwise combinations of niclosamide and chloroquine. After that, the cells were infected with SARS-CoV-2 at MOI 0.01 for 1 hour. The virus inoculum was discarded, and the cells were further maintained in the medium containing drugs for 2 days. The viral RNA was quantitated using one-step qRT-PCR. The dose-response curves of two-drug combination treatments against SARS-CoV-2 are shown; (**A**) serial dilutions of niclosamide in the presence of different fixed concentrations of chloroquine, (**B**) serial dilutions of chloroquine in the presence of different fixed concentrations of niclosamide. The synergy scores of two-drug combinations were calculated using SynergyFinderPlus. The dose-response matrix (**C**) and the Loewe synergy score map of two-drug combination treatment (**D**) are shown. The synergy scores less than − 10 accounted for the antagonistic effect; from − 10 to 10 accounted for the additive effect; and larger than 10 accounted for the synergistic effect between two drugs. The experiments were repeated at least three times, and data are shown as mean ± SD in **A**, **B** and **C** or mean [95% confidence intervals (CI)] in **D**

It was found that the presence of chloroquine induced a shift in niclosamide dose-response curve, with 3.308 and 1.483-fold reduction of niclosamide IC_50_ value in the presence of 0.425, and 0.2125 μM chloroquine, respectively (Fig. 3A, Table 2). A similar trend was observed for the chloroquine dose-response curve in the presence of niclosamide, with 3.57 and 1.68-fold reduction of chloroquine IC_50_ value in the presence of 0.0225 and 0.01125 μM niclosamide, respectively (Fig. 3B, Table 2). The dose-response matrix shows increasing inhibitory effect of the combination with higher concentrations of niclosamide and chloroquine (Fig. 3C). From Fig. 3D, most parts of a synergy score plot show negative to low positive synergy scores with a mean Loewe synergy score of 0.68, indicating an additive effect between niclosamide and chloroquine. Except for the combination of 0.045 μM niclosamide and 0.85 μM chloroquine that shows a peak synergy score of 20.11, 95% CI [12.15, 23.29], indicating a synergistic effect at these concentrations. Additionally, the synergy scores calculated using ZIP and Bliss independence reference models gave the values of − 2.72 and − 2.8, respectively, which similarly indicated the additive effect. The HSA model resulted in the synergy score of 10.23, which accounted for the small level in synergistic effect. No significant cytotoxicity in all 16 pairwise combinations (Fig. 3A, B).

### Ivermectin-chloroquine combination

**Fig. 4:**
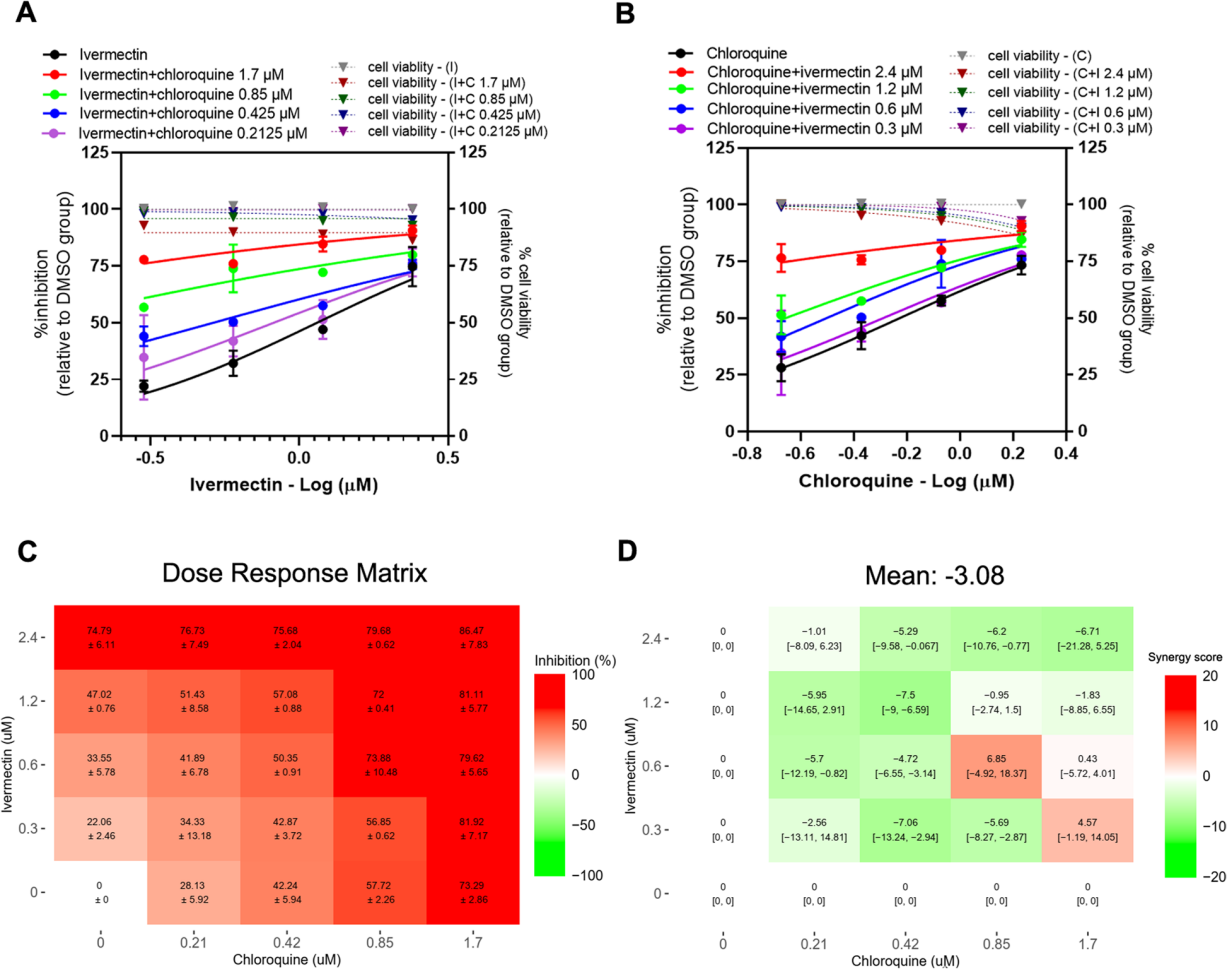
Ivermectin-chloroquine combination treatments against SARS-CoV-2 in Vero E6 cells. Vero E6 cells were treated for 1 hour with 16 different pairwise combinations of ivermectin and chloroquine. After that, the cells were infected with SARS-CoV-2 at MOI 0.01 for 1 hour. The virus inoculum was discarded, and the cells were further maintained in the medium containing drugs for 2 days. The viral RNA was quantitated using one-step qRT-PCR. The dose-response curves of two-drug combination treatments against SARS-CoV-2 are shown; (**A**) serial dilutions of ivermectin in the presence of different fixed concentrations of chloroquine, (**B**) serial dilutions of chloroquine in the presence of different fixed concentrations of ivermectin. The synergy scores of two-drug combinations were calculated using SynergyFinderPlus. The dose-response matrix (**C**) and the Loewe synergy score map of two-drug combination treatment (**D**) are shown. The synergy scores less than − 10 accounted for the antagonistic effect; from − 10 to 10 accounted for the additive effect; and larger than 10 accounted for the synergistic effect between two drugs. The experiments were repeated at least three times, and data are shown as mean ± SD in **A**, **B** and **C** or mean [95% confidence intervals (CI)] in **D**

The results showed that the presence of chloroquine induced a shift in ivermectin dose-response curve, with 2.47, 1.55-fold reduction of ivermectin IC_50_ value in the presence of 0.425, and 0.2125 μM chloroquine, respectively (Fig. 4A, Table 2). Similarly, the presence of ivermectin also induced a shift in chloroquine dose-response curve, with 2.83 and 1.73-fold reduction of chloroquine IC_50_ value in the presence of 0.6 and 0.3 μM ivermectin, respectively (Fig. 4B, Table 2). The dose-response matrix shows increasing inhibitory effect with higher concentrations of ivermectin and chloroquine (Fig. 4C). Most parts of a synergy score plot show negative synergy scores except for a peak positive score of 6.85, 95% CI [− 4.92, 18.37] in the combination of the 0.6 μM ivermectin and 0.85 μM chloroquine (Fig. 4D). The peak negative synergy score is − 7.5. As all of the different combinations had Loewe synergy scores between − 10 and 10 with a mean score of − 3.08, it suggests an additive effect between ivermectin and chloroquine. Moreover, ZIP, Bliss independence and HSA reference models showed the synergy scores of − 7.61, − 7.66 and 6.66, respectively, which indicated the additive effect. No significant cytotoxicity in all 16 pairwise combinations (Fig. 4A, B).

### Evaluation of single drug treatment against SARS-CoV-2 in Calu-3 cells

**Fig. 5:**
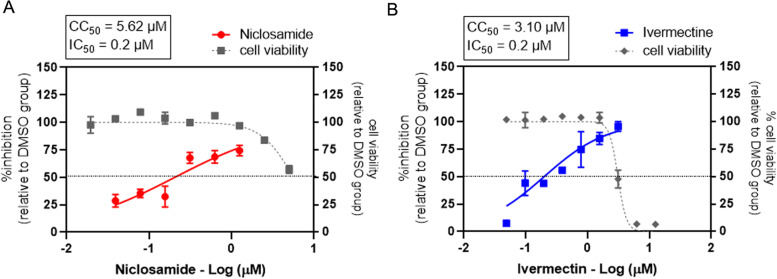
Single drug treatment against SARS-CoV-2 in Calu-3 cells. The dose-response curves of a single drug treatment against SARS-CoV-2 in Calu-3 cells are shown; (**A**) niclosamide, and (**B**) ivermectin. Calu-3 cells were treated with twofold serial dilutions of drug for 1 hour and followed by SARS-CoV-2 infection at 500 TCID_50_. Then the cells were maintained in the medium containing drugs or 0.5%DMSO for two days. Virus titers were determined using the plaque assay. The dose-response curves were expressed as the percent inhibition in relative to DMSO-treated cell control. The cell viability was determined using MTT assay and is expressed in relative to the DMSO-treated cell control. The experiments were repeated at least three times, and data are shown as mean ± SD

The best antiviral activity and calculated synergy scores demonstrated in the treatment with niclosamide-ivermectin combination in Vero E6 cells. Therefore, this two-drug combination was selected for the further evaluation in the human lung cancer cell line, Calu-3. The antiviral activities of single niclosamide and ivermectin treatments were assessed in Calu-3 cells (Fig. 5). The IC_50_ values of both drugs were 0.2 μM in Calu-3 cells. The CC_50_ values of niclosamide and ivermectin were 5.62 μM and 3.10 μM, respectively. The SI values of niclosamide and ivermectin were 28.1 and 15.5, respectively.

### Evaluation of Niclosamide-ivermectin combination treatment against SARS-CoV-2 in Calu-3 cells

**Fig. 6:**
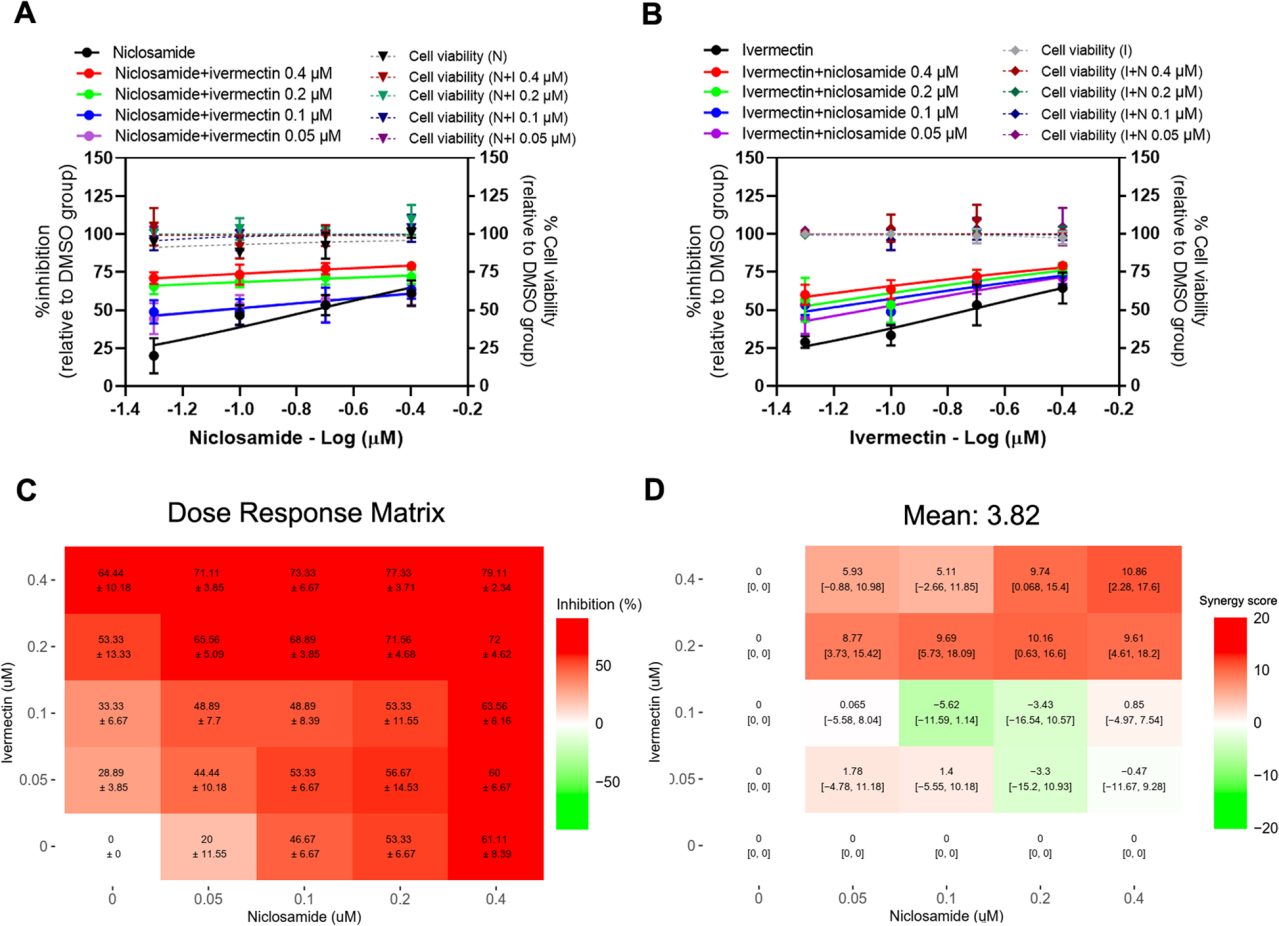
Niclosamide-Ivermectin combination treatments against SARS-CoV-2 in Calu-3 cells. Calu-3 cells were treated for 1 hour with 16 different pairwise combinations of niclosamide and ivermectin. After that, the cells were infected with SARS-CoV-2 at 500 TCID_50_ for 1 hour. The virus inoculum was discarded, and the cells were further maintained in the medium containing drugs for 2 days. The viral titers were determined using the plaque assay. The dose-response curves of two-drug combination treatments against SARS-CoV-2 are shown; (**A**) serial dilutions of niclosamide in the presence of different fixed concentrations of ivermectin, (**B**) serial dilutions of ivermectin in the presence of different fixed concentrations of niclosamide. The synergy scores of two-drug combinations were calculated using SynergyFinderPlus. The dose-response matrix (**C**) and the Loewe synergy score map of two-drug combination treatment (**D**) are shown. The synergy scores less than − 10 accounted for the antagonistic effect; from − 10 to 10 accounted for the additive effect; and larger than 10 accounted for the synergistic effect between two drugs. The experiments were repeated at least three times, and data are shown as mean ± SD in **A**, **B** and **C** or mean [95% confidence intervals (CI)] in **D**

The strong shifts were observed in the dose-response curves of niclosamide combined with 0.4 and 0.2 μM ivermectin (Fig. 6A). The presence of 0.1 and 0.05 μM ivermectin also induced a shift in the dose-response curve of niclosamide, with 2.38 and 2.33-fold reduction of niclosamide IC_50_ values, respectively (Fig. 6A, Table 3). In a similar way, the presence of niclosamide induced a shift in ivermectin dose-response curve with 3.64, and 2.41-fold reduction of ivermectin IC_50_ value in the presence of 0.1 and 0.05 μM niclosamide, respectively (Fig. 6B, Table 3). The dose–response matrix shows the increasing antiviral activity compared to the single drug treatments (Fig. 6C). The combination synergy analysis showed the mean Loewe synergy score of 3.82, which accounted for the additive effect between niclosamide and ivermectin in Calu-3 cells (Fig. 6D). Additionally, a peak Loewe synergy score of 10.86 showed in the combination of niclosamide and ivermectin at the highest concentrations (0.4 μM for both drugs). The synergy score obtained from ZIP, Bliss independence and HSA reference models were − 7.09, − 7.34 and 8.12, respectively, which also accounted for the additive effect between niclosamide and ivermectin. All 16 pairwise combinations showed no significant cytotoxicity (Fig. 6A, B).

**Table 3:** Evaluation of niclosamide-ivermectin combination treatments against SARS-CoV-2 in Calu-3 cells

Drug treatment	IC_**50**_ (μM)	Fold reduction of IC_**50**_ (single/combined)
**Niclosamide-ivermectin**		
**Niclosamide**	**0.20**	
Niclosamide + ivermectin 0.4 μM	ND	ND
Niclosamide + ivermectin 0.2 μM	ND	ND
Niclosamide + ivermectin 0.1 μM	0.084	2.38
Niclosamide + ivermectin 0.05 μM	0.086	2.33
**Ivermectin**	**0.20**	
Ivermectin + niclosamide 0.4 μM	ND	ND
Ivermectin + niclosamide 0.2 μM	ND	ND
Ivermectin + niclosamide 0.1 μM	0.055	3.64
Ivermectin + niclosamide 0.05 μM	0.083	2.41

*ND* not determined, cannot calculate IC_50_ with the least curve fit of the data sets

## Discussion

Our study shows that the repurposed anti-parasitic drugs, niclosamide, ivermectin and chloroquine possess high in vitro activity against SARS-CoV-2 as the IC_50_ values are in the low micromolar range. These results of single drugs treatments are in agreement with the previous studies [7, 14, 18, 26].

Niclosamide showed broad-spectrum antiviral activity against a wide range of viruses such as SARS-CoV [19, 27, 28], MERS-CoV [29], Zika virus [30], hepatitis C virus [31], Ebola virus [32] and Human immunodeficiency virus type 1 (HIV-1) [23]. The evidence found in other viruses suggested the plausible mechanisms of niclosamide in SARS-CoV-2 inhibition by blocking of viral entry via altering endosomal pH and the prevention of autophagy that led to the inhibition of virus replication [29, 33, 34]. Although niclosamide was originally thought to act on parasitic worms in the gut lumen and is barely absorbed to the blood stream, it was tested for various systemic repurposed treatments, and a maximal plasma concentration ranged from 35.7 to 182 ng ml^− 1^ (corresponding to 0.11–0.56 μM) was observed in a pharmacokinetic study [35–38]. This level exceeds the in vitro niclosamide IC_50_ against SARS-CoV-2, especially when used in the tested combinations. However, there have been little clinical data on niclosamide in COVID-19 treatment.

Chloroquine inhibits a broad range of viruses by blocking viral entry via inhibition of endosomal acidification [39]. It was recently shown that chloroquine could not inhibit SARS-CoV-2 in human lung cells because of the expression of TMPRSS2 [40]. This may at least partially explain the lack of clinical efficacy of this drug. Despite these in vitro anti-SARS-CoV-2 activities, clinical application of this drug to COVID-19 treatment has not yet been successful [8].

Previous in vitro studies suggested that ivermectin inhibits host importin alpha/beta-1 nuclear transport proteins, thus preventing the viruses from suppressing the host antiviral response [41]. Several studies reported antiviral activity of ivermectin on other viruses such as Zika virus [42], dengue virus [43–45], HIV-1 [46] and influenza A viruses [47]. Recently, it was found that ivermectin may interfere with the attachment of SARS-CoV-2 spike protein to the ACE2 receptor on human cell membrane [48]. Various possible mechanisms of action of ivermectin against SARS-CoV-2 had been proposed in both direct action on SARS-CoV-2 and host cellular targets [49]. However, uncertain clinical trial results varying from effective to no significant benefits were found in COVID-19 treatment using ivermectin [9–13, 50–53]. Oral administration of ivermectin (200 μg/kg) in humans resulted in a maximum plasma concentration at 0.049 ± 0.02 μM (mean ± SD) [54], which was lower than the in vitro IC_50_ values for anti-SARS-CoV-2 activity (IC_50_ Vero E6 = 1.23 μM, Calu-3 = 0.2 μM). However, higher levels of ivermectin were found in other tissues including fat, skin, and nodular tissues [55]. Different lung tissue concentrations of ivermectin were reported. The predicted maximum lung concentration calculated based on lung: plasma ratio in cattle was around twofold of the maximum plasma concentration (0.0873 μM) [56, 57]. However, a previous study in animals reported that the concentration of ivermectin in lung tissue may be 20 times higher than the plasma concentration [58], which exceeds the in vitro IC_50_ values for anti-SARS-CoV-2 activity. Moreover, ivermectin was shown to reduce the level of plasma nonstructural protein 1 in dengue patients, even though it showed high in vitro IC_50_ values against dengue virus (approximately 4.64 and 5.33 μM in Huh-7 cells and immortalized hepatocyte-like cell line) [59, 60]. This suggested that ivermectin tissue levels may be much higher than in plasma and may reach therapeutic antiviral level.

The lack of obvious clinical efficacy suggests that either these in vitro activities could not take effect in vivo*,* or the activities may not be sufficiently potent. An obvious strategy to enhance the potency is drug combination. While combining direct acting antivirals with different targets almost always results in additive or synergistic effect, combining drugs that act on host machineries does not always cause a synergistic effect and can even result in an antagonistic effect [26, 61]. Selecting proper drug combinations with synergistic effect is therefore crucial for development of efficacious regimens. In this study, two-drug combinations improve anti-SARS-CoV-2 activity as the greater viral inhibitory effects were observed with lower IC_50_ values compared to individual drugs. Although all combinations resulted in comparable levels of IC_50_ reduction indicating additive/synergy effect at levels lower than IC_50_ of individual drugs, only niclosamide-ivermectin resulted in enhanced activity at most of higher concentrations resulting in almost complete inhibition (> 99%) at the concentrations of about 2 times of IC_50_ of single drugs. At these concentrations, the single drugs could achieve only 70–80% inhibition. The enhanced combine-activity at both lower and higher concentrations resulted in the higher mean synergy score as compared to other combinations.

Our data may be useful in guiding the design of clinical trials that may generate a badly needed efficacious regimen for COVID-19 treatment and prevention. Although only the original strain was tested in this study, we do not expect the inhibitory effect to be much different among strains as the drugs target either the more conserved part of the virus or host machineries. Nevertheless, before attempting to use these drugs in clinical trials, the sensitivity of circulating viral strains should be confirmed.

## Conclusions

In conclusion, our study demonstrated the benefit of combining ivermectin, niclosamide and chloroquine on their anti-SAR-CoV-2 activities. Among the combinations, ivermectin and niclosamide showed the best synergistic profile. This combination should be further tested in clinical trials.

## Supplementary Information

**Additional file 1.**

## Data Availability

All data generated or analyzed during this study are included in this published article and its supplementary information files.

## Abbreviations

CC_50_: 50% cytotoxic concentration
Ct: Threshold cycle
DMEM/F-12: Dulbecco’s Modified Eagle Medium/Nutrient Mixture F-12
FBS: Fetal bovine serum
HSA: Highest Single Agent
IC_50_: Half maximal inhibitory concentration
Loewe: Loewe additivity
MEM: Minimum essential medium
MOI: Multiplicity of infection
PBS: Phosphate-buffered saline
Pfu/ml: Plaque forming units per ml
qRT-PCR: Quantitative reverse-transcription PCR
TCID_50_: 50% tissue culture infectious dose
TMPRSS2: Transmembrane Serine Protease 2
ZIP: Zero Independence Potency

## References

[1] Dyer O (2021). Covid-19: study claims real global deaths are twice official figures. BMJ.

[2] Mathieu E, Ritchie H, Ortiz-Ospina E, Roser M, Hasell J, Appel C, Giattino C, Rodés-Guirao L (2021). A global database of COVID-19 vaccinations. Nat Hum Behav.

[3] FDA Approves First Treatment for COVID-19. https://www.fda.gov/news-events/press-announcements/fda-approves-first-treatment-covid-19. Accessed 15 Dec 2021.

[4] Coronavirus (COVID-19) Update: FDA Authorizes Drug Combination for Treatment of COVID-19. https://www.fda.gov/news-events/press-announcements/coronavirus-covid-19-update-fda-authorizes-drug-combination-treatment-covid-19. Accessed 1 Dec 2021.

[5] Wang Y, Chen L (2020). Tissue distributions of antiviral drugs affect their capabilities of reducing viral loads in COVID-19 treatment. Eur J Pharmacol.

[6] Arshad U, Pertinez H, Box H, Tatham L, Rajoli RKR, Curley P, Neary M, Sharp J, Liptrott NJ, Valentijn A (2020). Prioritization of anti-SARS-CoV-2 drug repurposing opportunities based on plasma and target site concentrations derived from their established human pharmacokinetics. Clin Pharmacol Ther.

[7] Jeon S, Ko M, Lee J, Choi I, Byun SY, Park S, Shum D, Kim S (2020). Identification of antiviral drug candidates against SARS-CoV-2 from FDA-approved drugs. Antimicrob Agents Chemother.

[8] Singh B, Ryan H, Kredo T, Chaplin M, Fletcher T (2021). Chloroquine or hydroxychloroquine for prevention and treatment of COVID-19. Cochrane Database Syst Rev.

[9] Ahmed S, Karim MM, Ross AG, Hossain MS, Clemens JD, Sumiya MK, Phru CS, Rahman M, Zaman K, Somani J (2021). A five-day course of ivermectin for the treatment of COVID-19 may reduce the duration of illness. Int J Infect Dis.

[10] Behera P, Patro BK, Singh AK, Chandanshive PD, Ravikumar SR, Pradhan SK, SSK P, Batmanabane G, Mohapatra PR, Padhy BM (2021). Role of ivermectin in the prevention of SARS-CoV-2 infection among healthcare workers in India: a matched case-control study. PLoS One.

[11] Chaccour C, Casellas A, Blanco-Di Matteo A, Pineda I, Fernandez-Montero A, Ruiz-Castillo P, Richardson MA, Rodríguez-Mateos M, Jordán-Iborra C, Brew J (2021). The effect of early treatment with ivermectin on viral load, symptoms and humoral response in patients with non-severe COVID-19: a pilot, double-blind, placebo-controlled, randomized clinical trial. EClinicalMedicine.

[12] Lima-Morales R, Méndez-Hernández P, Flores YN, Osorno-Romero P, Cuecuecha-Rugerio E, Nava-Zamora A, Hernández-Galdamez DR, Romo-Dueñas DK, Salmerón J (2021). Effectiveness of a multidrug therapy consisting of ivermectin, azithromycin, montelukast and acetylsalicylic acid to prevent hospitalization and death among ambulatory COVID-19 cases in Tlaxcala, Mexico. Int J Infect Dis.

[13] Kory P, Meduri GU, Varon J, Iglesias J, Marik PE (2021). Review of the emerging evidence demonstrating the efficacy of Ivermectin in the prophylaxis and treatment of COVID-19. Am J Ther.

[14] Caly L, Druce JD, Catton MG, Jans DA, Wagstaff KM (2020). The FDA-approved drug ivermectin inhibits the replication of SARS-CoV-2 in vitro. Antivir Res.

[15] Jans DA, Wagstaff KM (2021). The broad spectrum host-directed agent ivermectin as an antiviral for SARS-CoV-2 ?. Biochem Biophys Res Commun.

[16] Elalfy H, Besheer T, El-Mesery A, El-Gilany A-H, Abd Elazez MS, Alhawarey A, et al. Effect of a combination of nitazoxanide, ribavirin, and ivermectin plus zinc supplement (MANS.NRIZ study) on the clearance of mild COVID-19. J Med Virol. 2021;93(5):3176–83. 10.1002/jmv.26880 PMC8014583 33590901

[17] Remme J, De Sole G, Dadzie KY, Alley ES, Baker RH, Habbema JD, Plaisier AP, van Oortmarssen GJ, Samba EM (1990). Large scale ivermectin distribution and its epidemiological consequences. Acta Leiden.

[18] Mostafa A, Kandeil A, AMME Elshaier Y, Kutkat O, Moatasim Y, Rashad AA, Shehata M, Gomaa MR, Mahrous N, Mahmoud SH (2020). FDA-approved drugs with potent *in vitro* antiviral activity against severe acute respiratory syndrome coronavirus 2. Pharm J.

[19] Xu J, Shi P-Y, Li H, Zhou J (2020). Broad Spectrum antiviral agent Niclosamide and its therapeutic potential. ACS Infect Dis.

[20] Kanjanasirirat P, Suksatu A, Manopwisedjaroen S, Munyoo B, Tuchinda P, Jearawuttanakul K, Seemakhan S, Charoensutthivarakul S, Wongtrakoongate P, Rangkasenee N (2020). High-content screening of Thai medicinal plants reveals Boesenbergia rotunda extract and its component Panduratin a as anti-SARS-CoV-2 agents. Sci Rep.

[21] Reed LJ, Muench H (1938). A simple method of estimating fifty percent enpoint. Am J Epidemiol.

[22] Ganguly D, Rottet S, Yee S, Hee W, Smith A, Khin N, Millar A, Fahrer A. SYBR green one-step qRT-PCR for the detection of SARS-CoV-2 RNA in saliva. bioRxiv. 2020:05.29.109702. 10.1101/2020.05.29.109702.

[23] Niyomdecha N, Suptawiwat O, Boonarkart C, Jitobaom K, Auewarakul P (2020). Inhibition of human immunodeficiency virus type 1 by niclosamide through mTORC1 inhibition. Heliyon.

[24] Zheng S, Wang W, Aldahdooh J, Malyutina A, Shadbahr T, Tanoli Z, Pessia A, Tang J (2022). SynergyFinder plus: toward better interpretation and annotation of drug combination screening datasets. Genomics Proteomics Bioinformatics.

[25] Loewe S (1953). The problem of synergism and antagonism of combined drugs. Arzneimittelforschung.

[26] Pizzorno A, Padey B, Dubois J, Julien T, Traversier A, Dulière V, Brun P, Lina B, Rosa-Calatrava M, Terrier O (2020). In vitro evaluation of antiviral activity of single and combined repurposable drugs against SARS-CoV-2. Antivir Res.

[27] Wen C-C, Kuo Y-H, Jan J-T, Liang P-H, Wang S-Y, Liu H-G, Lee C-K, Chang S-T, Kuo C-J, Lee S-S (2007). Specific plant terpenoids and lignoids possess potent antiviral activities against severe acute respiratory syndrome coronavirus. J Med Chem.

[28] Wu C-J, Jan J-T, Chen C-M, Hsieh H-P, Hwang D-R, Liu H-W, Liu C-Y, Huang H-W, Chen S-C, Hong C-F (2004). Inhibition of severe acute respiratory syndrome coronavirus replication by Niclosamide. Antimicrob Agents Chemother.

[29] Gassen NC, Niemeyer D, Muth D, Corman VM, Martinelli S, Gassen A, Hafner K, Papies J, Mösbauer K, Zellner A (2019). SKP2 attenuates autophagy through Beclin1-ubiquitination and its inhibition reduces MERS-coronavirus infection. Nat Commun.

[30] Xu M, Lee EM, Wen Z, Cheng Y, Huang WK, Qian X, Tcw J, Kouznetsova J, Ogden SC, Hammack C (2016). Identification of small-molecule inhibitors of Zika virus infection and induced neural cell death via a drug repurposing screen. Nat Med.

[31] Stachulski AV, Pidathala C, Row EC, Sharma R, Berry NG, Lawrenson AS, Moores SL, Iqbal M, Bentley J, Allman SA (2011). Thiazolides as novel antiviral agents. 2. Inhibition of hepatitis C virus replication. J Med Chem.

[32] Madrid PB, Panchal RG, Warren TK, Shurtleff AC, Endsley AN, Green CE, Kolokoltsov A, Davey R, Manger ID, Gilfillan L (2015). Evaluation of Ebola virus inhibitors for drug repurposing. ACS Infect Dis.

[33] Thomson G (2020). COVID-19: social distancing, ACE 2 receptors, protease inhibitors and beyond?. Int J Clin Pract.

[34] Jurgeit A, McDowell R, Moese S, Meldrum E, Schwendener R, Greber UF (2012). Niclosamide is a proton carrier and targets acidic endosomes with broad antiviral effects. PLoS Pathog.

[35] Schweizer MT, Haugk K, McKiernan JS, Gulati R, Cheng HH, Maes JL, Dumpit RF, Nelson PS, Montgomery B, McCune JS (2018). A phase I study of niclosamide in combination with enzalutamide in men with castration-resistant prostate cancer. PLoS One.

[36] Andrews P, Thyssen J, Lorke D (1982). The biology and toxicology of molluscicides, Bayluscide. Pharmacol Ther.

[37] Li Y, Li PK, Roberts MJ, Arend RC, Samant RS, Buchsbaum DJ (2014). Multi-targeted therapy of cancer by niclosamide: a new application for an old drug. Cancer Lett.

[38] Burock S, Daum S, Keilholz U, Neumann K, Walther W, Stein U (2018). Phase II trial to investigate the safety and efficacy of orally applied niclosamide in patients with metachronous or sychronous metastases of a colorectal cancer progressing after therapy: the NIKOLO trial. BMC Cancer.

[39] Savarino A, Boelaert JR, Cassone A, Majori G, Cauda R (2003). Effects of chloroquine on viral infections: an old drug against today’s diseases?. Lancet Infect Dis.

[40] Hoffmann M, Mösbauer K, Hofmann-Winkler H, Kaul A, Kleine-Weber H, Krüger N, Gassen NC, Müller MA, Drosten C, Pöhlmann S (2020). Chloroquine does not inhibit infection of human lung cells with SARS-CoV-2. Nature.

[41] Yang SNY, Atkinson SC, Wang C, Lee A, Bogoyevitch MA, Borg NA, Jans DA (2020). The broad spectrum antiviral ivermectin targets the host nuclear transport importin α/β1 heterodimer. Antivir Res.

[42] Barrows NJ, Campos RK, Powell ST, Prasanth KR, Schott-Lerner G, Soto-Acosta R, Galarza-Muñoz G, McGrath EL, Urrabaz-Garza R, Gao J (2016). A screen of FDA-approved drugs for inhibitors of Zika virus infection. Cell Host Microbe.

[43] Wagstaff KM, Sivakumaran H, Heaton SM, Harrich D, Jans DA (2012). Ivermectin is a specific inhibitor of importin α/β-mediated nuclear import able to inhibit replication of HIV-1 and dengue virus. Biochem J.

[44] Tay MY, Fraser JE, Chan WK, Moreland NJ, Rathore AP, Wang C, Vasudevan SG, Jans DA (2013). Nuclear localization of dengue virus (DENV) 1-4 non-structural protein 5; protection against all 4 DENV serotypes by the inhibitor Ivermectin. Antivir Res.

[45] Xu T-L, Han Y, Liu W, Pang X-Y, Zheng B, Zhang Y, Zhou X-N (2018). Antivirus effectiveness of ivermectin on dengue virus type 2 in Aedes albopictus. PLoS Negl Trop Dis.

[46] Wagstaff KM, Rawlinson SM, Hearps AC, Jans DA (2011). An AlphaScreen®-based assay for high-throughput screening for specific inhibitors of nuclear import. J Biomol Screen.

[47] Götz V, Magar L, Dornfeld D, Giese S, Pohlmann A, Höper D, Kong B-W, Jans DA, Beer M, Haller O, Schwemmle M (2016). Influenza a viruses escape from MxA restriction at the expense of efficient nuclear vRNP import. Sci Rep.

[48] Lehrer S, Rheinstein PH (2020). Ivermectin docks to the SARS-CoV-2 spike receptor-binding domain attached to ACE2. In Vivo.

[49] Zaidi AK, Dehgani-Mobaraki P (2022). The mechanisms of action of ivermectin against SARS-CoV-2—an extensive review. J Antibiot.

[50] Popp MSM, Metzendorf MI, Gould S, Kranke P, Meybohm P, Skoetz N, Weibel S (2021). Ivermectin for preventing and treating COVID-19. Cochrane Database Syst Rev.

[51] Vallejos J, Zoni R, Bangher M, Villamandos S, Bobadilla A, Plano F, Campias C, Chaparro Campias E, Medina MF, Achinelli F (2021). Ivermectin to prevent hospitalizations in patients with COVID-19 (IVERCOR-COVID19) a randomized, double-blind, placebo-controlled trial. BMC Infect Dis.

[52] Lim SCL, Hor CP, Tay KH, Mat Jelani A, Tan WH, Ker HB, Chow TS, Zaid M, Cheah WK, Lim HH (2022). Efficacy of Ivermectin treatment on disease progression among adults with mild to moderate COVID-19 and comorbidities: the I-TECH randomized clinical trial. JAMA Intern Med.

[53] Bryant A, Lawrie TA, Dowswell T, Fordham EJ, Mitchell S, Hill SR, Tham TC (2021). Ivermectin for prevention and treatment of COVID-19 infection: a systematic review, meta-analysis, and trial sequential analysis to inform clinical guidelines. Am J Ther.

[54] Muñoz J, Ballester MR, Antonijoan RM, Gich I, Rodríguez M, Colli E, Gold S, Krolewiecki AJ (2018). Safety and pharmacokinetic profile of fixed-dose ivermectin with an innovative 18mg tablet in healthy adult volunteers. PLoS Negl Trop Dis.

[55] Baraka OZ, Mahmoud BM, Marschke CK, Geary TG, Homeida MM, Williams JF (1996). Ivermectin distribution in the plasma and tissues of patients infected with Onchocerca volvulus. Eur J Clin Pharmacol.

[56] Schmith VD, Zhou J, Lohmer LRL (2020). The approved dose of Ivermectin alone is not the ideal dose for the treatment of COVID-19. Clin Pharmacol Ther.

[57] Lifschitz A, Virkel G, Sallovitz J, Sutra JF, Galtier P, Alvinerie M, Lanusse C (2000). Comparative distribution of ivermectin and doramectin to parasite location tissues in cattle. Vet Parasitol.

[58] Chiu SHL, Green ML, Baylis FP, Eline D, Rosegay A, Meriwether H, Jacob TA (1990). Absorption, tissue distribution, and excretion of tritium-labeled ivermectin in cattle, sheep, and rat. J Agric Food Chem.

[59] Kongmanas K, Punyadee N, Wasuworawong K, Songjaeng A, Prommool T, Pewkliang Y, Manocheewa S, Thiemmeca S, Sa-Ngiamsuntorn K, Puttikhunt C (2020). Immortalized stem cell-derived hepatocyte-like cells: an alternative model for studying dengue pathogenesis and therapy. PLoS Negl Trop Dis.

[60] Suputtamongkol Y, Avirutnan P, Mairiang D, Angkasekwinai N, Niwattayakul K, Yamasmith E, Saleh-Arong FA, Songjaeng A, Prommool T, Tangthawornchaikul N (2021). Ivermectin accelerates circulating nonstructural protein 1 (NS1) clearance in adult dengue patients: a combined phase 2/3 randomized double-blinded placebo controlled trial. Clin Infect Dis.

[61] Bobrowski T, Chen L, Eastman RT, Itkin Z, Shinn P, Chen CZ, Guo H, Zheng W, Michael S, Simeonov A (2021). Synergistic and antagonistic drug combinations against SARS-CoV-2. Mol Ther.

